# Phytofabrication of Selenium Nanoparticles with *Moringa oleifera* (MO-SeNPs) and Exploring Its Antioxidant and Antidiabetic Potential

**DOI:** 10.3390/molecules28145322

**Published:** 2023-07-10

**Authors:** Anas Ahzaruddin Ahamad Tarmizi, Nik Nasihah Nik Ramli, Siti Hajar Adam, Maisarah Abdul Mutalib, Mohd Helmy Mokhtar, Shirley Gee Hoon Tang

**Affiliations:** 1 School of Graduate Studies (SGS), Management and Science University, Shah Alam 40100, Malaysia; anasahzaruddin@gmail.com (A.A.A.T.); maisarah_abdulmutalib@msu.edu.my (M.A.M.); 2 Pre-Clinical Department, Faculty of Medicine and Defence Health, Universiti Pertahanan Nasional Malaysia, Kuala Lumpur 57000, Malaysia; 3 Department of Physiology, Faculty of Medicine, Universiti Kebangsaan Malaysia, Kuala Lumpur 56000, Malaysia; helmy@ukm.edu.my; 4 Center for Toxicology and Health Risk Studies (CORE), Faculty of Health Sciences, Universiti Kebangsaan Malaysia, Kuala Lumpur 50300, Malaysia; shirleytgh@ukm.edu.my

**Keywords:** selenium nanoparticles, green synthesis, *Moringa oleifera*, optimization, antioxidant, antidiabetic

## Abstract

The advancement in nanotechnology is the trigger for exploring the synthesis of selenium nanoparticles and their use in biomedicine. Therefore, this study aims to synthesize selenium nanoparticles using *M. oleifera* as a reducing agent and evaluate their antioxidant and antidiabetic potential. Our result demonstrated a change in the color of the mixture from yellow to red, and UV-Vis spectrometry of the suspension solution confirmed the formation of MO-SeNPs with a single absorbance peak in the range of 240–560 nm wavelength. FTIR analysis revealed several bioactive compounds, such as phenols and amines, that could possibly be responsible for the reduction and stabilization of the MO-SeNPs. FESEM + EDX analysis revealed that the amorphous MO-SeNPs are of high purity, have a spherical shape, and have a size of 20–250 nm in diameter, as determined by HRTEM. MO-SeNPs also exhibit the highest DPPH scavenging activity of 84% at 1000 μg/mL with an IC50 of 454.1 μg/mL and noteworthy reducing ability by reducing power assay. Furthermore, MO-SeNPs showed promising antidiabetic properties with dose-dependent inhibition of α-amylase (26.7% to 44.53%) and α-glucosidase enzyme (4.73% to 19.26%). Hence, these results demonstrated that *M. oleifera* plant extract possesses the potential to reduce selenium ions to SeNPs under optimized conditions with notable antioxidant and antidiabetic activities.

## 1. Introduction

Selenium (Se) is a mineral trace element commonly discovered in nature [1]. Eswayah et al. (2016) discussed that the biomethylation of elemental selenium in the atmosphere by environmental microorganisms followed by rainfall contributes to the distribution of selenium in soil and groundwater, which is subsequently taken up by plants and animals [2]. On the other hand, organically bound selenium is present in animals in the form of proteins containing selenocysteine amino acid residues, known as selenoproteins. Since the discovery of the hepatoprotective properties of selenium in 1957, more research has been done to explore the biological mechanisms of selenium’s effect on human health and metabolism [3]. The significant role of selenium in normal physiological functions has been clearly emphasized by extensive research that has found a link between selenium deficiency and infertility, thyroid disorders, cardiomyopathy, Kaschin-Beck disease, Keshan disease, cardiovascular diseases, and Alzheimer’s disease [4,5].

Most selenoproteins are functionally characterized as co-factors of enzymes involved in redox reactions and preventing the formation of free radicals and reactive oxygen species in the cell. The enzymes include (i) glutathione peroxidases (GPx), which neutralize intracellular and intercellular hydrogen peroxide (H_2_O_2_) and organic peroxides, (ii) thioredoxin reductases (TrxR), which, together with thioredoxin, reduce nicotinamide-adenine-dinucleotide-phosphate (NADPH), which is responsible for the reduction of disulphide bonds and the regulation of mitochondrial H_2_O_2_ emission, (iii) iodothyronine deiodinidases, which act in the activation and deactivation of thyroid hormones [6]. These antioxidant properties of selenium led to a strong interest in the scientific community to explore its therapeutic potential in chronic diseases caused by oxidative stress, such as diabetes mellitus, cardiovascular diseases, and autoimmune diseases [7]. When metabolized, the redox reactions exerted by lower molecular weight seleno-organic compounds can even influence molecular processes of DNA repair and epigenetics [8].

Nanoparticles (NPs) are known as ultrafine particles with dimensions between 1 and 100 nm. In the medical field, nanotechnology has been hailed as the holy grail of drug delivery systems. The use of nanoparticles can improve bioavailability and receptor specificity, thereby reducing the frequency of administration, toxicity, and severe side effects of the drug [9]. Similarly, selenium nanoparticles (SeNPs) have demonstrated less toxicity and greater biocompatibility than their organic or inorganic forms [10]. There are several approaches for the synthesis of nanoparticles, of which the green synthesis method using plants for the bio-reduction of the compound in nanomaterials is the best option [11]. This is because the production of nanoparticles via green synthesis has high compatibility, lower toxicity, environmental friendliness, and cost efficiency compared to the physical and chemical approaches [11].

*Moringa oleifera* (*M. oleifera*) is an evergreen, deciduous, medium-sized plant native to South Asia. The entire plant of *M. oleifera* can be utilized, with each part of the tree being a major source of various bioactive compounds such as flavonoids, polyphenols, glucosinolates, tocopherols, carotenoids, folate, and polyunsaturated fatty acids [12]. The plant is known for its antidiabetic, antipyretic, antitumor, anti-inflammatory, antihypertensive, hepatoprotective, diuretic, antioxidant, antimicrobial, and cardio-stimulant properties. Globally, this plant is widely used as an alternative medicine for home remedies to treat inflammation and allergies [13]. The prominent pharmacological activities of *M. oleifera* combined with the richness of bioactive compounds in this plant suggest that it could have a reducing agent for the synthesis of SeNPs. The synergy between SeNPs and *M. oleifera* could have tremendous bioactivity potential that could be exploited in various pharmacological fields. Therefore, the present study aims to optimize and characterize SeNPs in a green synthesis approach using *M. oleifera* as a reducing agent and to evaluate their antioxidant and antidiabetic potential.

## 2. Results

### 2.1. Biogenic Synthesis and Optimization of MO-SeNPs

**Figure 1:**
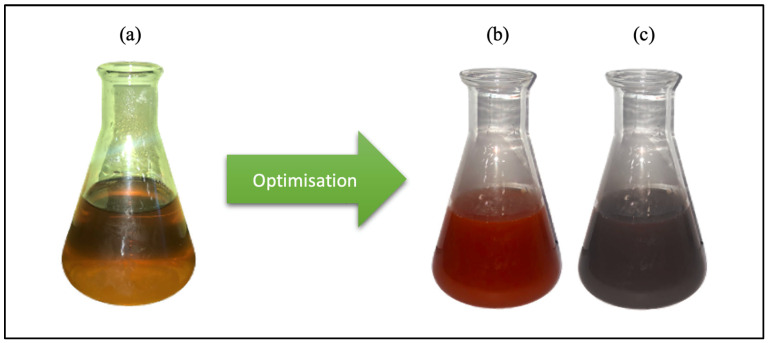
Color changes of the mixture from green synthesis after the optimization procedure. (**a**) *M. oleifera* plant extract + sodium selenite solution; (**b**) Red SeNPs; (**c**) Black SeNPs.

During the optimization process, two different color changes were recorded: yellow to red or yellow to black (Figure 1). Optimization of MO-SeNPs synthesis was observed in all groups by reading the surface plasmon resonance (SPR) band and absorbance peak with the UV-Vis spectrophotometer. The UV-Vis spectra showed that the absorbance was between 400 nm and 520 nm, with a single peak for all optimization groups.

### 2.2. Effects of pH Level on MO-SeNPs Synthesis

**Figure 2:**
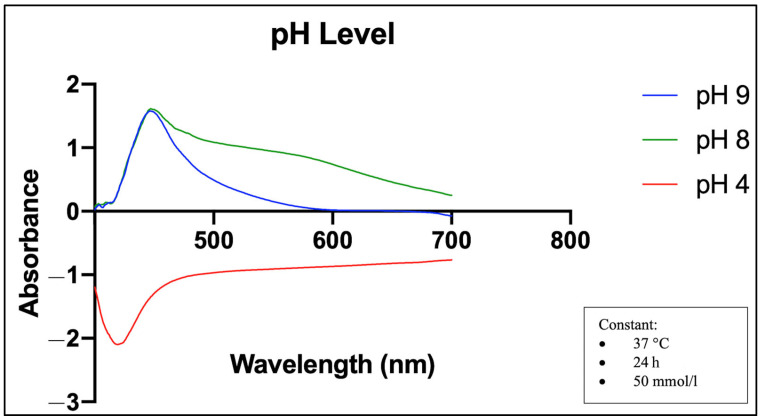
SPR absorption spectra of UV-Vis at different pH levels of synthesis. The natural state without alteration = pH 8, plant extract = pH 6. Temperature (37 °C), incubation time (24 h), and selenite concentration (50 mmol/L) were kept constant.

Figure 2 shows that the UV-Vis spectra for three pH values show a positive absorbance peak under alkaline conditions (pH 8 and 9). The SPR band at pH 8 was relatively broader than the SPR band at pH 9, but both bands recorded the same peak at 445 nm. However, under acidic conditions (pH 4), the SPR band showed a negative absorbance result.

### 2.3. Effects of Incubation Time on MO-SeNPs Synthesis

**Figure 3:**
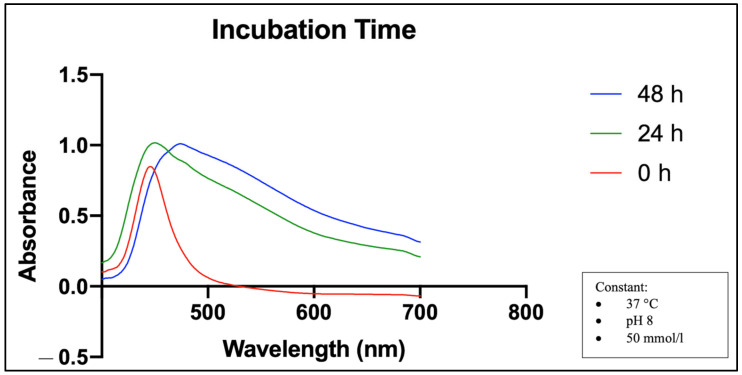
SPR absorption spectra of UV-Vis at different incubation times. Temperature (37 °C), pH level (pH 8), and selenite concentration (50 mmol/L) were kept constant.

Figure 3 shows that the UV-Vis spectra at the beginning of the synthesis (0 h) recorded an absorbance peak at 446 nm with a slightly lower absorbance value than after 24 and 48 h. Over the next 24 and 48 h, the absorbance peak increased in intensity and shifted to a longer wavelength. The optimization wavelength was measured at 450 nm after 24 h and at 474 nm after 48 h.

### 2.4. Effects of Temperature on MO-SeNPs Synthesis

**Figure 4:**
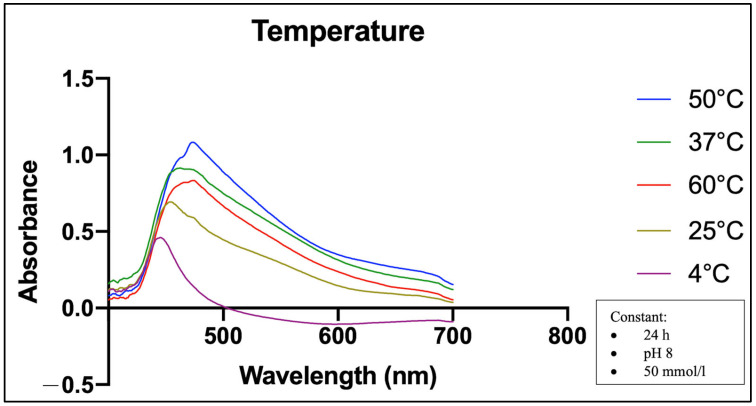
SPR absorption spectra of UV-Vis at different incubation temperatures of the synthesis. Incubation time (24 h), pH level (pH 8), and selenite concentration (50 mmol/L) were kept constant.

Figure 4 displays that the UV-Vis spectra of five temperatures have some differences in their absorbance level and wavelength intensity. The results show that 4 °C has the lowest absorbance and the shortest wavelength (446 nm), followed by 25 °C (453 nm) compared to the other temperatures. The absorbance and wavelength increased significantly with increasing temperatures. The longest wavelength intensity of the MO-SeNPs was shown at 50 °C and 60 °C, both of which had a peak at 474 nm. However, the absorbance level at 60 °C is much lower than at 50 °C in the first stage and 37 °C in the second stage. The wavelength of the SeNPs at 37 °C was measured at 461 nm.

### 2.5. Effects of Precursor Concentration on MO-SeNPs Synthesis

**Figure 5:**
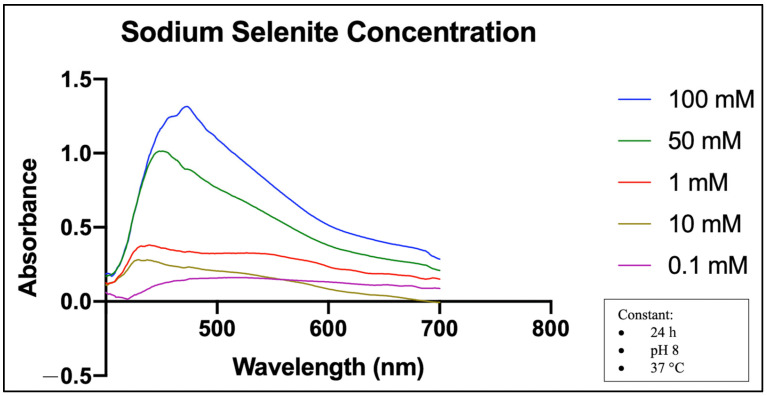
SPR absorption spectra of UV-Vis at different sodium selenite concentrations. mM = mmol/L. Incubation time (24 h), pH level (pH 8), and temperature (37 °C) were kept constant.

In Figure 5, the UV-Vis spectra of MO-SeNPs at five precursor concentrations show that the highest concentration of sodium selenite (100 mM) produces the highest absorbance, with the peak at 474 nm wavelength, followed by 50 mM at 450 nm wavelength. The lowest concentration, which is 0.1 mM, has the lowest absorbance, with a peak at 520 nm. At a concentration of 1 mM of precursor, the absorbance is slightly higher and the peak is at 441 nm, compared to a concentration of 10 mM, but at a shorter wavelength of 431 nm.

### 2.6. Characterizations of MO-SeNPs

Characterization is required to evaluate the material characteristics and microscopic configuration and to further verify the arrangement and structure of the substances and materials in the synthesized products [14]. In this study, the five samples with the highest UV-Vis absorbance were selected, as higher absorbance may indicate a higher proportion of SeNPs in the mixture. Further characterization was performed using energy dispersive X-ray analysis (EDX) to identify the elemental composition of the MO-SeNPs samples [15].

#### 2.6.1. EDX Spectra Analysis

**Figure 6:**
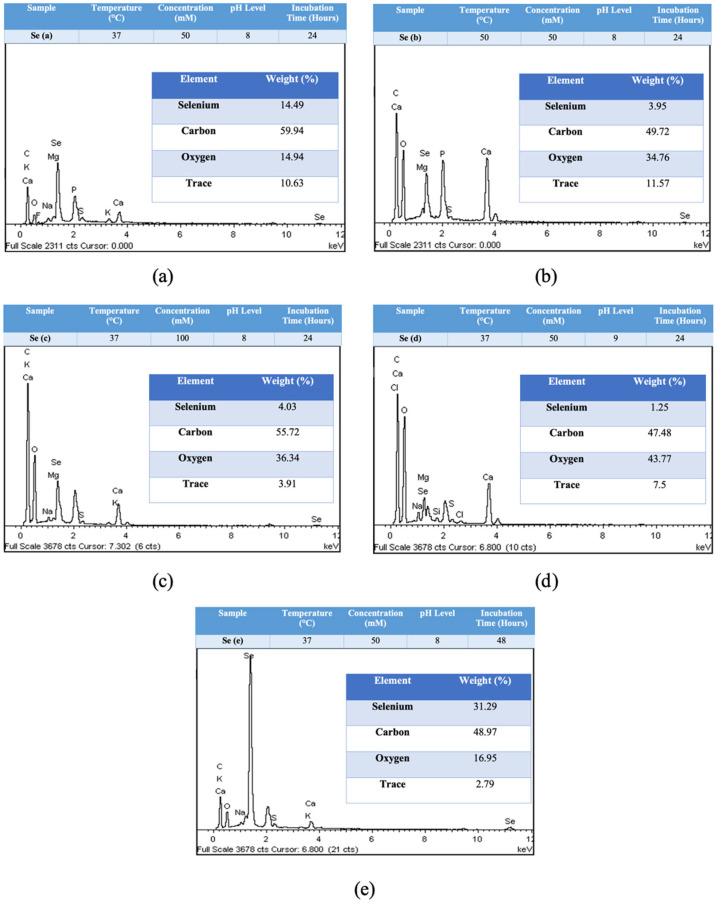
EDX spectra reading of MO-SeNPs from five samples with different optimisation parameters (**a**–**e**) as stated on each spectra. Se = selenium, C = carbon, O = oxygen, K = potassium, Ca = calcium, F = fluorine, Na = sodium, Mg = magnesium, P = phosphate, S = sulfur, keV = kiloelectron volt.

Figure 6 shows that the presence of a peak at 1.4 keV in the EDX spectra confirms the presence of elemental Se (SeLα) in all of the samples [16]. However, the individual samples had different intensities and weight percentages, with sample Se (e) having the highest weight of 31.29%. In some studies, a higher weight percentage of selenium was reported, e.g., 50% and 60% [17,18]. Therefore, sample Se (e) with the highest selenium composition was chosen for further characterization using Fourier Transform Infrared (FTIR) analysis.

#### 2.6.2. FTIR Analysis

**Figure 7:**
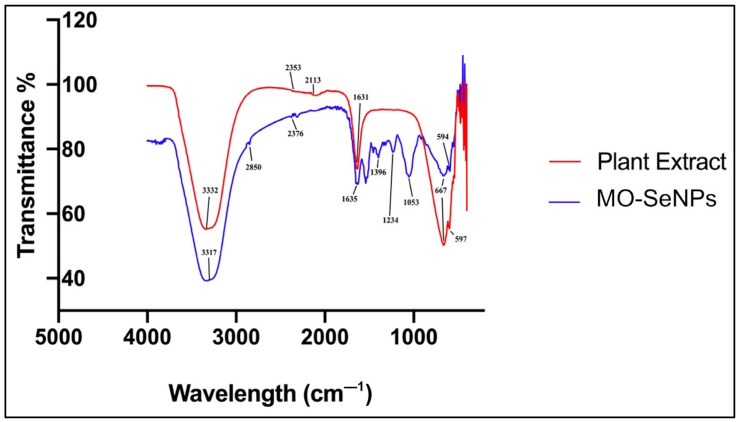
FTIR spectra of *M. oleifera* plant extract and MO-SeNPs. Transmittance peaks were detected at 3332, 2353, 2113, 1631, 667, and 597 cm^−1^ for the *M. oleifera* extract and at 3317, 2850, 2376, 1635, 1396, 1234, 1053, 667, and 597 cm^−1^ for the MO-SeNPs.

FTIR analyses were performed to identify the responsible constituents from plant extract that act as reducing agents in the synthesis of MO-SeNPs and as capping agents to prevent aggressive agglomeration [19]. FTIR analysis of both materials showed multiple peaks throughout the spectrum, and the results are presented in Figure 7.

#### 2.6.3. Field Emission Scanning Electron Microscopy (FESEM) & High Resolution Transmission Electron Microscopy (HRTEM) Analysis

**Figure 8:**
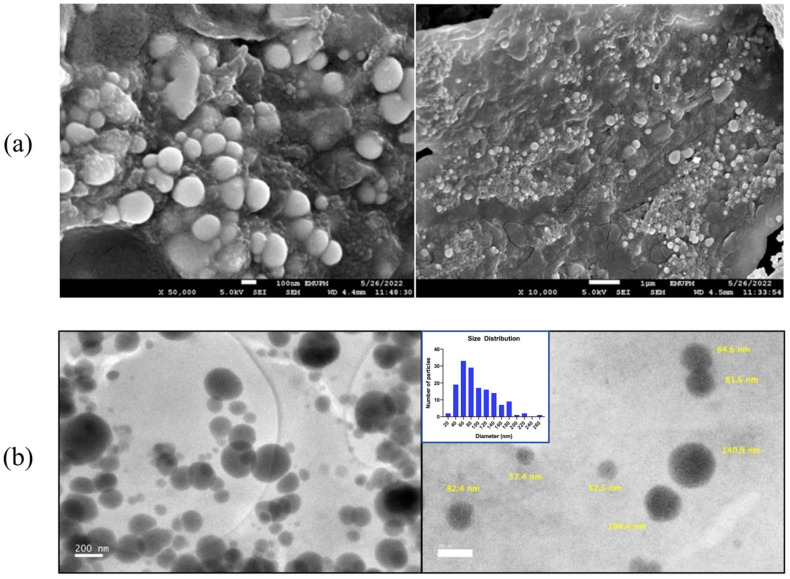
(**a**) FESEM imaging of MO-SeNPs (**b**) HRTEM imaging of MO-SeNPs.

To verify the size and morphology of the synthesized MO-SeNPs, FESEM and HRTEM analyses of sample Se (e) were also performed. The high magnification of the FESEM image in Figure 8a shows that the MO-SeNPs have an almost spherical shape with a smooth surface. The FESEM image also shows that the MO-SeNPs are well distributed in powder form with slight agglomeration. Figure 8b shows that the diameter of the MO-SeNPs is in the range of 20–250 nm. Moreover, more than half of the MO-SeNPs diameters were recorded to be smaller than 100 nm, with a mean of 95.36 nm. Therefore, the optimization parameters from sample Se (e) were used as the optimal synthesis conditions for MO-SeNPs and applied for their antioxidant and antidiabetic potential.

### 2.7. In-Vitro Antioxidant Assays

**Figure 9:**
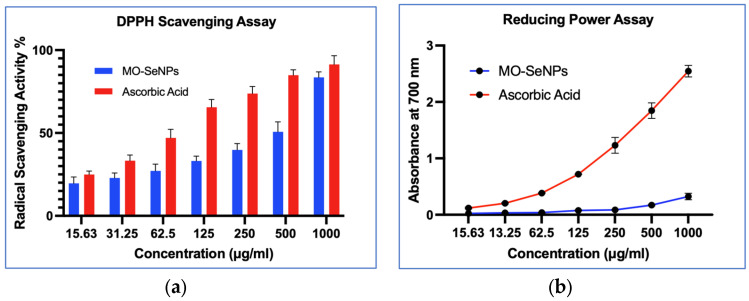
(**a**) DPPH scavenging assay; (**b**) Reducing power assay.

In this study, the antioxidant potential of the synthesized MO-SeNPs was determined by a DPPH radical scavenging assay and a reducing power assay (Figure 9).

#### 2.7.1. DPPH Radical Scavenging Activity

DPPH (2,2-diphenyl-1-picrylhydrazyl) is a stable compound that accepts electrons from synthesized Se nanoparticles. The efficiency of the MO-SeNPs was observed by the color shift from purple to yellow and quantified with a spectrophotometer. We found that the MO-SeNPs exhibited concentration-dependent DPPH scavenging activity at concentrations of 15.63–1000 μg/mL. The highest concentration used in this study (1000 μg/mL) showed 84% scavenging activity, which is comparable to that of ascorbic acid, which is 91% at the same dose. The IC_50_, which is the minimum concentration to scavenge the DPPH free radical by 50% [20], was 454.1 μg/mL and 111.8 μg/mL, respectively.

#### 2.7.2. Reducing Power Assay

The reducing power assay of the MO-SeNPs and ascorbic acid was evaluated by the reduction of the Fe^3+/^ferricyanide complex to the ferrous form. The Fe^2+^ complex was measured by the formation of Perl’s Prussian blue at 700 nm. As can be seen in Figure 9, the reducing power of the MO-SeNPs was concentration-dependent, with the peak absorbance at the highest concentration used in this study, 1000 μg/mL, being 0.324 compared to 2.247 for ascorbic acid used as a control. Despite the concentration-dependent increase, the degree of reducing the power of the MO-SeNPs was lower compared to ascorbic acid.

The IC_50_ value for the reducing power assay expressed as an exact concentration provided an absorbance of 0.5 at 700 nm [21]. In this study, the IC_50_ for the reducing power assay of ascorbic acid and MO-SeNPs was recorded at 74.4 µg/mL and 1597.9 µg/mL, respectively.

### 2.8. In-Vitro Antidiabetic Assays

The antidiabetic potential of MO-SeNPs was evaluated by studying the inhibition of carbohydrate-digesting enzymes, namely α-amylase and α-glucosidase. α-Amylase plays a role in regulating blood glucose levels by catalyzing the conversion of polysaccharides by cleaving α-1,4-glycosidic bonds into smaller oligosaccharide pieces, which are later broken down by α-glucosidase. The α-glucosidase further hydrolyzes the terminal, non-reducing 1,4-linked α-glucose residues to release absorbable monosaccharides into the bloodstream [22]. Inhibition of these enzymes could be one of the measures to control the postprandial rise in blood glucose levels.

#### 2.8.1. α-Amylase Inhibition Activity

In this study, MO-SeNPs showed a concentration-dependent increase in the inhibitory activity of the enzyme α-amylase, with the highest inhibition observed at a dose of 1000 μg/mL, 44.53%. The inhibitor control provided showed a strong inhibitory activity of α-amylase (94.5%) at 100 μg/mL.

#### 2.8.2. α-Glucosidase Inhibition Activity

**Figure 10:**
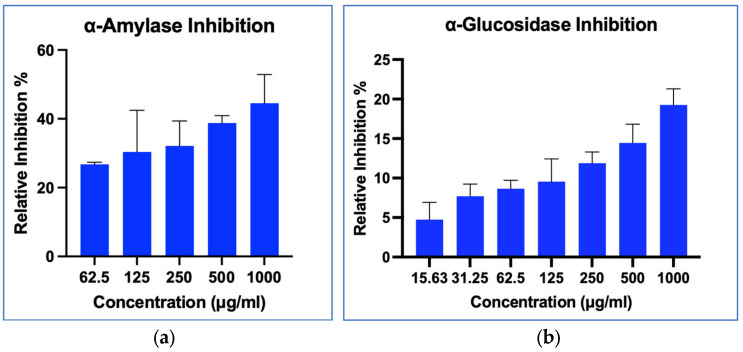
(**a**) α-Amylase inhibition assay; (**b**) α-Glucosidase inhibition assay.

The inhibitory effect of MO-SeNPs on the enzyme α-glucosidase was observed to be concentration-dependent, as depicted in Figure 10. The highest relative inhibition achieved in this study was 19.26% at a concentration of 1000 μg/mL. In comparison, the control group treated with acarbose exhibited strong enzyme inhibition, reaching 91.3% at a concentration of 100 μg/mL.

## 3. Discussion

### 3.1. Biogenic Synthesis and Optimisation of MO-SeNPs

NPs can be synthesized by physical, chemical, and biological methods [23]. The physical method belongs to the top-down approach, while the chemical and biological methods belong to the bottom-up approach [24]. Compared to physical and chemical methods, biological synthesis is more environmentally friendly because the physical method releases energy and heat into the atmosphere, while the chemical method usually produces toxic and hazardous substances as by-products that can affect the environment [25]. In the biological method, or green synthesis, of NPs, biological organisms such as microorganisms or plants are used as reducing agents. Among these options, plant-mediated synthesis has greater potential than microbial routes because it does not require the complex laboratory preparations and cell culture resources that are essential for microorganism-mediated synthesis. The microbial source for the synthesis of SeNPs may have some limitations that need to be addressed, such as the need for a sterile environment, a time-consuming process due to microbial growth, and other necessities of specific microbial species and strains [26]. Plants, as the main source for the synthesis of SeNPs, are also abundant and filled with various potential phytochemical constituents that can act as both reducing and stabilizing agents. Plant-mediated synthesis is considered to be inexpensive, low in toxicity, and environmentally friendly compared to other methods of synthesis of SeNPs [27].

In this study, the leaves of *M. oleifera* were used as the main plant for the green synthesis of SeNPs. *M. oleifera* was found to contain various active metabolites such as polyphenols, alkaloids, carotenoid compounds, terpenoids, and sulphur [28]. To obtain the active constituents of the plant, an aqueous extraction of the leaves of *M. oleifera* was prepared for this study. Several studies on the green synthesis of SeNPs have shown that aqueous extraction is used with various plants such as *Emblica officinalis*, *Withania somnifera* (Ashwaganda), and *Ceropegia bulbosa* [17,19,29].

The synthesis of SeNPs mediated by *M. oleifera* leaf extracts was visually observed by the change in color of the mixture. Initially, the *M. oleifera* leaf extract was yellow, which turned brick red or black after the addition of sodium selenite solution. The change in color of the solution indicates the reduction of sodium selenite into the element selenium. The observed reduction potential could be due to the effect of phenols and flavonoids extracted from *M. oleifera* [30].

Figure 1 confirms the successful synthesis of SeNPs based on the color changes after mixing the *M. oleifera* plant extract with the sodium selenite solution. The color changes were due to the increase in SeNP concentration in the synthesis mixture. The two different colors (red and black) indicate different forms of elemental selenium being synthesized. The amorphous selenium appears red, while the vitreous selenium is gray [31]. The vitreous selenium must be distinguished as unstable SeNPs, which appear black after synthesis because unstable SeNPs continuously aggregate into a stable but inactive element, which affects its bioactivity [32].

So far, some optimization techniques such as response surface methodology (RSM) and central composite rotatable design (CCRD) have been applied for the analysis of a response that can be influenced by several parameters [33,34]. However, the optimization method of MO-SeNPs in this study was performed according to the method of Wen et al. (2021), as it involves a similar sample of SeNPs [15]. For preliminary confirmation of MO-SeNPs synthesis, the SPR band and absorption peak were observed by a UV-Vis spectrophotometer. The UV-Vis spectra revealed that the absorption range of the MO-SeNPs is between 400 and 520 nm, with a single peak in all optimization groups. This result is in agreement with a previous report where a similar absorption range between 400 and 600 nm wavelength was observed for the synthesized SeNPs with citrus extracts [35]. Similarly, Borah et al. [36] reported that the synthesis of SeNPs using *Bacillus paramycoides* showed an absorbance peak between 400 and 580 nm.

Optimization of SeNPs synthesis using plant extracts has already been performed with selenium, silver, and copper oxide nanoparticles but is usually done by microbially mediated synthesis [37,38]. For this study, four optimization parameters were evaluated, including pH, incubation temperature, incubation period, and precursor concentration [39,40]. These parameters were selected based on optimization reports from previous studies on the synthesis of SeNPs with plant extracts. The incubation pressure and the volume of the extract also influence the synthesis of SeNPs but were not considered in this study [41,42].

SeNPs synthesized with *M. oleifera* showed only a single peak during the entire optimization period. Previous studies reported that for spherical or nearly spherical nanoparticles, the absorption peak appears as a single SPR resonance, while anisotropic shapes show more than one SPR resonance depending on their shape [43]. However, the appearance of more than one peak may also indicate that there is a different population of SeNPs with different sizes or that an organic molecule has been detected in the compound [44,45].

In this study, the effect of pH was tested with three pH values by changing strong bases (sodium hydroxide) and strong acids (hydrochloric acid). The synthesis mixture, without any pH change, showed a slightly alkaline value at pH 8 after the addition of sodium selenite. This is because sodium selenite (Na_2_SeO_3_) undergoes hydrolysis after the addition of water and produces sodium hydroxide (NaOH) as a by-product. The chemical reaction can be expressed as follows:

Na_2_SeO_3_ + 2H_2_O = H_2_SeO_3_ + 2NaOH

Figure 2 shows that the SPR band at a pH of 8 was relatively broad compared to the SPR band at a pH of 9. A previous study noted that a broader peak could indicate a relatively wider size distribution of particles and that there are more negative hydroxide ions (OH-) in a higher pH medium, which would enhance the reduction activity [46].

The effect of incubation time in Figure 3 shows that the absorption peak increased in intensity and shifted to a longer wavelength over the next 24 and 48 h. These two changes were also observed for other optimization parameters such as precursor concentration and temperature in the synthesis of MO-SeNPs (see Figure 4 and Figure 5).

Wen et al. [15] stated that the increase in the absorption peak indicates an increase in the number of particles in the mixture, and the wavelength shift indicates an increase in the particle size. This fact is due to the fact that smaller particles with higher surface energy require more energetic light with a shorter wavelength to produce plasma resonance, while larger particles work in the opposite way [47]. Thus, this study suggests that the amount of synthesized SeNPs increased proportionally with increasing incubation time, sodium selenite concentration, and incubation temperature. Interestingly, we found that the absorption peaks were lower at an incubation temperature of 60 °C than at 50 °C and 37 °C, as shown in Figure 4. This could indicate a decrease in the bioactive components of the synthesis mixture at 60 °C. A previous study also reported that incubation with hot air at 60 °C leads to a decrease in phenols in the aqueous extract and some alcohol extracts [48].

### 3.2. Characterization of MO-SeNPs

In a previous report, the presence of other elements in EDX spectral analysis was found to be due to the presence of biomaterial as a capping agent on the surface of the nanoparticles [16]. In addition, the smaller size of the nanoparticles requires a larger amount of stabilizing molecules on the surface of the particles [49]. This report is in agreement with the present study, as EDX analysis showed the presence of other elements such as oxygen and carbon. This could indicate that the capping agent present on the surface of MO-SeNPs consists of carbon and oxygen atoms. Bioactive compounds such as amino and carbonyl groups in plant extracts are thought to play a vital role in the encapsulation of nanoparticles by reducing surface free energy and stabilizing the nanoparticles [15].

The FTIR result showed a broad peak at 3332 cm^−1^ in the plant extract and a shift at 3317 cm^−1^ in MO-SeNPs corresponding to the O–H stretch of the hydroxyl group and phenols. This result suggests the possibility that phenolic compounds are the major reducing agents in the synthesis of the MO-SeNPs in this study. The FTIR band peak at 1635 cm^−1^ in the plant extract and shift to 1631 cm^−1^ in MO-SeNPs corresponds to the C = O stretch of the amide in the carbonyl group. The spectral peak at 667 cm^−1^ in both spectra corresponds to the C–Br stretch of the aliphatic bromo compound [50]. Constituents such as polyphenols and vitamin C contain multiple hydroxyl groups (–OH), which have a strong reducing ability and can reduce selenite ions (Se^+4^) to elemental selenium atoms (Se^0^). Furthermore, the presence of a double-bonded carbonyl compound in the analysis can support the role of the functional carbonyl group, which can act as a stabilizing agent. This is because selenium atoms have a high surface energy, which leads to the aggregation of numerous atoms into nanoparticles to reduce the surface energy. The aggregation of selenium atoms also results in the formation of a complex bond with bioactive macromolecules, which form a protective layer for the nanoparticles and reduce the surface energy, resulting in stable SeNPs [15].

Electron micrographs from FESEM and HRTEM showed some agglomeration of the MO-SeNPs. Agglomerated nanoparticles were believed to have higher biological potential in previous analyses [19]. In support of this study, SeNPs synthesized with Aloe vera leaf extract and Okra extract were found to have SeNPs sizes of 50 nm and 46 nm in diameter, respectively [51,52]. On the other hand, studies with hawthorn fruit extract and walnut leaf extract showed larger average sizes of SeNPs at 113 nm and 150 nm in diameter, respectively [53,54]. Meanwhile, Gunti et al. [17] found that the size of nanoparticles depends on the active compound of the biomaterial used.

### 3.3. In-Vitro Antioxidant Assays

Antioxidants are stable molecules that are able to donate an electron to a charged free radical and neutralize it to prevent the radical from causing damage. Antioxidants can act as radical scavengers, hydrogen donors, electron donors, peroxide decomposers, singlet oxygen quenchers, enzyme inhibitors, synergists, and metal chelators [55].

In this study, the antioxidant activity of the MO-SeNPs was evaluated using DPPH for scavenging activity as well as its reducing capacity. These activities were compared with ascorbic acid, a potent antioxidant agent. Our result shows that MO-SeNPs exhibit concentration-dependent DPPH scavenging activity. This result is consistent with previous studies using SeNPs prepared from *Spirulina platensis*, which showed a DPPH scavenging activity of 89% at a concentration of 500 μg/mL, and another study using SeNPs from *Tinospora cordifolia*, which showed a scavenging activity of 78% at 100 μg/mL [56,57].

However, the MO-SeNPs IC_50_ value obtained in this study was higher than that of ascorbic acid. The higher IC_50_ values suggest that a higher concentration of the compound is required to scavenge the radicals, resulting in lower scavenging activity but still having great potential as free radical scavengers. Qiu et al. [58] also reported an almost similar IC_50_ value of 500 μg/mL in their pectin-stabilized SeNPs.

Compared to the antioxidant scavenging effect, MO-SeNPs exhibit weaker Fe^3+^/ferricyanide complex reduction capacity. The maximum absorbance of MO-SeNPs was recorded at 0.324, while ascorbic acid recorded a value of 2.247 at 700 nm with a concentration of 1000 μg/mL. In contrast, Cao et al. [59] reported a better maximum absorbance of 3, using SeNPs synthesized with the seaweed species *Grateloupia filicina* at a dose of 150 μg/mL. Our findings suggest that a higher concentration of MO-SeNPs is required to exert a reducing effect.

Based on this understanding, the synthesized MO-SeNPs were more potent in their antioxidant activity in scavenging DPPH radicals than in reducing ferric ions. This result could be due to the presence of hydroxyl and carbonyl groups on the surface of the MO-SeNPs, which contribute to the transfer of hydrogen atoms (HAT) [60]. In addition to the electron- or hydrogen-donating property, the size of the synthesized SeNPs was also a decisive factor in determining the antioxidant potential. Smaller particles with a larger surface area had a considerable number of reactive sites for free radicals, resulting in better antioxidant capacity [18]. Therefore, modulating the size of synthetic SeNPs could greatly contribute to enhancing the antioxidant effect. In contrast, Sentkowska et al. [61] suggest that particle homogeneity has a greater impact on the antioxidant capacity of NPs than their size. Similarly, aggregation or agglomeration, especially in aqueous solutions, could reduce the antioxidant potential of MO-SeNPs. Therefore, maintaining the stability of the synthesized MO-SeNPs could further enhance their antioxidant potential either through scavenging or reducing activity.

### 3.4. In-Vitro Antidiabetic Assays

In this study, different concentrations of the synthesized MO-SeNPs were prepared to evaluate their inhibitory effects on α-amylase and α-glucosidase enzymes. The inhibitory effect of the MO-SeNPs was compared with acarbose, one of the clinically available drugs to inhibit the carbohydrate digestive enzyme, and with *Triticum aestivum*, commonly known as common wheat, which has been shown to have promising inhibition of the α-amylase enzyme [62].

We found that the MO-SeNPs displayed dose-dependent inhibition of α-amylase, with the highest dosage showing 44.53% inhibition. This finding is in agreement with that of Tang et al. [18] who reported that SeNPs synthesized with *Gracilaria lemaneiformis* showed dose-dependent inhibition of α-amylase enzyme with an IC_50_ of 1.55 mg/mL. Similarly, Deepa et al. [63] reported dose-dependent α-amylase inhibition of SeNPs synthesized using *Cassica ariculata* flowers (CAF) as a reducing agent with 95% inhibition compared to acarbose at a concentration of 100 μg/mL. However, inhibition of α-amylase with MO-SeNPs may require a higher concentration than the dosage currently used in this study to achieve the IC_50_ value.

The different results observed in this study could be due to different methodologies, the concentration of the SeNPs, and the origin of the α-amylase used. Similar to antioxidant capacity, the size of the SeNPs could influence inhibitory enzyme activity. SeNPs synthesized with CAF with a diameter of 11 nm demonstrated more pronounced inhibition compared to SeNPs capped with seaweed polysaccharides, which had a diameter of 84 nm. Interestingly, our own synthesized SeNPs (MO-SeNPs) exhibited a size similar to the SeNPs capped with seaweed polysaccharides and displayed comparable inhibition percentages at a concentration of 1 mg/mL. These findings suggest that SeNPs size may serve as a contributing factor influencing the inhibition of carbohydrate enzymes [18,63].

However, the mild inhibitory activities on α-amylase could be beneficial, as strong inhibition of the α-amylase enzyme could lead to possible undesirable side effects. Stronger inhibition of the carbohydrate-digesting enzyme such as α-amylase may lead to incomplete digestion of the carbohydrates, which are eventually fermented by the colonic bacteria, leading to GI disturbances such as bloating, nausea, flatulence, and diarrhoea, to name a few [64].

Similar to α-amylase, a dose-dependent relative inhibitory effect on the α-glucosidase enzyme was observed in this study. This finding is in agreement with Prasathkumar et al. [65], Tang et al. [18], and Deepa et al. [63], although higher doses of SeNPs were used in their studies. However, similar to α-amylase inhibitory activity, the dosage used in this study is not high enough to reveal the IC_50_ value. The highest dosage of 1000 μg/mL showed only 19.26% of the relative inhibition percentage, suggesting that larger doses are needed to achieve this effect. Therefore, further studies need to be conducted to determine the optimal concentration without compromising the toxicity potential.

In addition, the molecular interactions between the enzyme and the SeNPs, such as van der Waals, electrostatic forces, hydrogen, and hydrophobic bonds, need to be further investigated to determine the mode of inhibition (competitive or non-competitive). Cha et al. [66] reported that the inhibitory effects observed in SeNPs could be due to van der Waals forces acting on the nanosized particles and causing a reduction in the carbohydrate-hydrolysing enzyme, which is more likely to lead to a non-competitive type of enzyme inhibition. Similarly, the reversibility of SeNPs enzyme binding could be further investigated before MO-SeNPs are marketed as a potential means of controlling postprandial blood glucose levels in diabetic patients.

## 4. Materials and Methods

### 4.1. Material

Sodium selenite (Na_2_SeO_3_) (≥98%) and 2,2-diphenyl-1-picrylhydrazyl (DPPH) were purchased from Sigma-Aldrich, Burlington, MA, USA. Sodium hydroxide (NaOH), hydrochloric acid (HCl), potassium ferricyanide (K_3_[Fe(CN)_6_]), ferric chloride (FeCl_3_), trichloroacetic acid (C_2_HCl_3_O_2_), phosphate buffer solution (PBS), and ascorbic acid were all analytically graded and obtained from R&M Chemicals, Subang, Malaysia.

### 4.2. Moringa oleifera

The leaves of *M. oleifera* were supplied by a local dealer from Sungai Petani, Kedah, Malaysia. Taxonomic identification was verified by a certified botanist from Universiti Putra Malaysia (Voucher No. MFI 0213/21). All leaves were carefully washed under running tap water in order to remove any contamination and dirt before being dried in a laboratory oven at 40 °C for three days. The dried leaves were later ground into a fine powder using an industrial blender, and the powder was stored in an airtight container at room temperature for further use.

### 4.3. Aqueous Extract Preparation

The aqueous extract of *M. oleifera* was prepared according to the previous study by García-Beltrán et al. [67] with slight modifications. 20 g of ground leaf powder of *M. oleifera* was homogenized with 800 mL of boiling distilled water using a conical flask for the maceration process. During maceration, the mixture was shaken for 4 h at room temperature with an orbital shaker at 150 rpm. The mixture was then centrifuged at 4000 rpm for 20 min before being filtered with Whatman No. 1 filter paper to remove the particulate matter completely. The filtered aqueous plant extract was stored in a sealed container at 4 °C for the preparation of the nanoparticles.

### 4.4. Green Synthesis of MO-SeNPs

A 50 mM sodium selenite solution was prepared by adding 86.47 mg sodium selenite powder to 10 mL of double-distilled water. Later, 5 mL of the sodium selenite solution was added dropwise to 20 mL of *M. oleifera* aqueous extract under magnetic stirring at room temperature. The stirring was maintained for 1 min, and the reaction mixture was continued with different optimization parameters.

### 4.5. Optimization of MO-SeNPs Synthesis

To synthesize the MO-SeNPs, the pH, temperature, incubation time, and concentration of the precursors were optimized according to the method of Wen et al. (2021) with some modifications [15]. The presence of the synthesized MO-SeNPs was detected by measuring the absorption peak using a UV-Vis spectrophotometer (Hach DR6000 UV-Vis Spectrophotometer, Ames, IA, USA) at a wavelength between 200 and 700 nm [68]. The synthesized MO-SeNPs pellet was then purified three times with double-distilled water and redispersed in a phosphate buffer solution before being lyophilized in a freeze dryer to obtain the powder form [15,69].

#### 4.5.1. pH Level

The sodium selenite-*M. oleifera* aqueous extract pH mixture was varied to three different pHs (pH 4, pH 8, and pH 9) with an accuracy of ±0.2. A suitable volume of 1 M of sodium hydroxide and 1 M of hydrochloric acid was used for the pH adjustment of the mixture.

#### 4.5.2. Temperature

To investigate the effect of incubation temperature on the synthesis of MO-SeNPs, the reaction mixture was incubated at five different temperatures (4 °C, 25 °C, 37 °C, 50 °C, and 60 °C) for 24 h in a laboratory incubator.

#### 4.5.3. Incubation Time

The effect of incubation time on the synthesis mixture was determined by observing the reaction mixture immediately (0 h) and after the incubation process for 24 h and 48 h.

#### 4.5.4. Precursor (Sodium Selenite) Concentration

Five sodium selenite concentrations (0.1 mM, 1 mM, 10 mM, 50 mM, and 100 mM) were prepared to identify the effect of precursor concentration on MO-SeNPs synthesis.

### 4.6. Characterization of MO-SeNPs

#### 4.6.1. Energy Dispersive X-ray (EDX)

Energy-dispersive X-ray (EDX) analysis was used to identify the elemental compositions of the synthesized nanoparticle. Five samples with the highest absorbance from the UV-Vis spectrometry results were chosen for EDX analysis since a higher absorbance may indicate a higher amount of SeNPs in the mixture for further characterization with EDX to identify the elemental composition of the MO-SeNPs samples [15].

#### 4.6.2. Fourier Transform Infrared (FTIR) Spectroscopy

A Fourier transform infrared spectrophotometer (Shimadzu IRAffinity-1, Kyoto, Japan) was used in order to identify the responsible phytochemical compounds with various functional groups that act as the reducing and capping agents in the aqueous leaf extract of *M. oleifera* and to study their potential role in the fabrication of MO-SeNPs. The MO-SeNPs solution was characterized by scanning the spectra in the range of 4000–400 cm^−1^ wavenumbers.

#### 4.6.3. Field Emission Scanning Electron Microscope (FESEM)

Field emission scanning electron microscopy (JEOL JSM-7600F, Tokyo, Japan) was performed to identify the surface morphology of the nanoparticles. The powder form of MO-SeNPs was coated with platinum using a sputter coater before the samples were filled into their holders. The MO-SeNPs were suspended in absolute ethanol, and single drops of the suspension were staged on the loading grid and dried for analysis.

#### 4.6.4. High Resolution Transmission Electron Microscopy (HRTEM)

High resolution transmission electron microscopy (JEOL JEM-F200, Tokyo, Japan) was executed to determine the size distribution and the exact shape of the MO-SeNPs. The MO-SeNPs were sonicated and set carefully on a copper coated grid to be viewed under the microscope.

### 4.7. In-Vitro Antioxidant Assays

#### 4.7.1. DPPH Radical Scavenging Activity

A DPPH radical scavenging assay was performed to evaluate the antioxidant potential of the synthesized MO-SeNPs using the method described by Hashem et al. [69] with slight modification. Briefly, two-fold serial dilutions were done to prepare different concentrations of SeNPs (1000, 500, 250, 125, 62.5, 31.25, and 15.63 μg/mL). 200 mL of each concentration was mixed individually with 800 μL of a 1 mM DPPH (2,2-diphenyl-1-picrylhydrazyl) solution in methanol and incubated for 30 min in the dark at room temperature. After incubation, the samples were centrifuged at 6000 rpm for 20 min, and the absorbance of the samples was measured at 517 nm using a UV-Vis spectrophotometer. The reaction mixture without MO-SeNPs was used as a control, and the procedure was repeated with ascorbic acid as the standard and performed in triplicate. The percentage of DPPH scavenging activity was calculated according to the following formula:

$$\frac{Absorbance\;Control-Absorbance\;Test}{Absorbance\;Control}\times 100$$

#### 4.7.2. Reducing Power Assay

The ferric reducing capacity of the synthesized MO-SeNPs was investigated with the potassium ferricyanide-ferric chloride method as described by Abdulmalek and Balbaa [70]. Several different concentrations of the synthesized MO-SeNPs and also ascorbic acid (1000, 500, 250, 125, 62.5, 31.25, 15.63 μg/mL) were prepared along with 2.5 mL of phosphate buffer solution (0.2 M, pH 6.6) and 2.5 mL of 1% potassium ferricyanide (K_3_[Fe(CN)_6_]) and incubated at 50 °C for 20 min to reduce ferricyanide into ferrocyanide. The reaction was halted by adding 2.5 mL of 10% (*w*/*v*) trichloroacetic acid (TCA), followed by centrifugation at 3000 rpm for 10 min. Finally, 2.5 mL of the supernatant was added to 0.5 mL of freshly prepared 0.1% ferric chloride (FeCl_3_). The procedure was repeated with ascorbic acid as the standard and performed in triplicate. The reducing power was determined by absorbance and observed at 700 nm with a spectrophotometer.

### 4.8. In-Vitro Antidiabetic Assays

#### 4.8.1. α-Amylase Inhibition Assay

The α-amylase inhibition assay was performed using an inhibition enzyme kit (ab283391) (Abcam, Cambridge, UK) [71]. The synthesised MO-SeNPs were diluted with different concentrations (1000, 500, 250, 125, and 62.5 μg/mL) and each concentration was further diluted 100-fold with distilled water and another 3-fold with assay buffer. A total of 50 μL of each sample was mixed with 100 μL α-amylase enzyme-substrate reaction mixture, and the optical density was observed at 405 nm in kinetic mode for 25 min with a 5 min interval during incubation at room temperature. Inhibitor control provided by the manufacturer was used as standard (*Triticum aestivum*, 100 mg/mL). The tests were performed in triplicate, and the percentage of relative inhibition of α-amylase was calculated using the formula:

$$\frac{Slope\;of\;Enzyme\;Control-Slope\;of\;Sample}{Slope\;of\;Enzyme\;Control}\times 100$$

#### 4.8.2. α-Glucosidase Inhibition Assay

The α-glucosidase inhibition test was performed according to the manufacturer’s stated protocol (ab284520) (Abcam, Cambridge, UK). Briefly, different concentrations of MO-SeNPs (1000, 500, 250, 125, 62.5, 31.25, and 15.63 μg/mL) were prepared and diluted 100-fold. 10 μL of the MO-SeNPs were added to 40 μL of the α-glucosidase enzyme reaction mixture and incubated for 60 min at room temperature. Optical density was observed at a wavelength of 410 nm for 60 min with an interval of 5 min, and acarbose (100 μg/mL) provided in the assay kit was used as a standard inhibitor control. All assays were performed in triplicate. The percentage of relative inhibition was calculated using the following formula:

$$\frac{Slope\;of\;Enzyme\;Control-Slope\;of\;Sample}{Slope\;of\;Enzyme\;Control}\times 100$$

### 4.9. Data Analysis

Data analysis of the results and images was done using Image J software (Version 1.53t). IC_50_ values were determined by linear regression analysis from the mean inhibition value. All the data acquired throughout the experimental period was recorded in Microsoft Excel. Recorded optimization results, antidiabetic assays, and antioxidant assay analysis were conducted using GraphPad Prism 9.

## 5. Conclusions

In conclusion, this study has shown that MO-SeNPs can be synthesized under optimized conditions using the plant extract of *M. oleifera*. The results indicate that the optimization of MO-SeNPs at an initial pH of 8, an incubation time of 48 h at 37 °C, and the use of a 50 mM sodium selenite solution gave the best results compared to other optimization parameters. The phenol-rich *M. oleifera* could be the main component responsible for the selenium ion reduction process. In the in vitro study of MO-SeNPs, despite the limitations encountered, MO-SeNPs succeeded in showing two biological effects. This study has shown that MO-SeNPs have a considerable radical scavenging effect and remarkable reducing power. In addition, the in vitro anti-diabetic assay has shown that MO-SeNPs can exhibit better inhibition of α-amylase than the α-glucosidase enzyme.

## Data Availability

All data related to this research are presented in the manuscript.
